# A Rare Case of Necrotizing Soft Tissue Infection in the Labium Resulting From Candida glabrata

**DOI:** 10.7759/cureus.92428

**Published:** 2025-09-16

**Authors:** Maria-Anthi Chantzi, Alexandra Marinou, Maria Papadoliopoulou, Theodoros Sidiropoulos, Nick Danias

**Affiliations:** 1 School of Medicine, National and Kapodistrian University of Athens, Athens, GRC; 2 4th Department of Surgery, Attikon University Hospital, National and Kapodistrian University of Athens, Athens, GRC

**Keywords:** candida glabrata, female, fournier gangrene, fungal necrotising soft tissue infections, non-albicans candida

## Abstract

Necrotizing soft tissue infections (NSTIs) are severe infections affecting the skin, subcutaneous, and muscle tissue. Tissue destruction, systemic toxicity, and increased morbidity are usually observed. An aggressive form of NSTI is called Fournier’s gangrene: it starts as a necrotizing fasciitis of the perineal and genital area and affects adjacent soft tissue. It exhibits an increased rate of morbidity, thus requiring a high index of clinical suspicion to secure early surgical debridement. A 58-year-old patient presented in the emergency department complaining of pain in the labia majora. Clinical examination revealed tenderness, erythema, edema, and the presence of a large condyloma on the left labium majus. The patient was admitted and was treated with an antibiotic, with no improvement. A crackling sensation of the affected labium was observed on subsequent examination. Imaging revealed signs of necrosis, and NSTI was confirmed. The patient underwent surgical debridement of the perineum along with removal of the condyloma. A vacuum-assisted closure device was used for secondary healing of the surgical wound. Culture of the excised tissue and purulent discharge resulted in the isolation of a rare causative agent of fungal Fournier’s gangrene, *Candida glabrata*. Thus, the patient’s antibiotic treatment was escalated, and antifungal treatment was initiated with anidulafungin. The patient was discharged on the 32nd postoperative day. Delay in diagnosis and initiation of surgical treatment for NSTIs is the most significant factor affecting morbidity, which ranges between 20% and 40%. Despite the nonspecific signs and symptoms that often lead to misdiagnosis, timely recognition and intervention are crucial and can lead to a favorable outcome, as in this case.

## Introduction

Fournier's gangrene is a rare, rapidly progressive, and life-threatening type of necrotizing fasciitis, affecting the superficial and deep tissues of the perineal, perianal, and genital regions. Due to the spreading inflammation and infection, blood vessel thrombosis occurs, resulting in ischemia and creating an environment conducive to further bacterial proliferation and production of toxins and destructive enzymes, ultimately leading to necrosis in soft tissues and fascia [1]. Abdominal involvement can also occur in cases where the Dartos, Colles, and Scarpa fasciae are affected [1]. Considering the high morbidity and mortality rate of the disease, early recognition and aggressive intervention are required [1].

The disease was named after Jean Alfred Fournier, a French dermatologist who initially described it as an idiopathic, rapidly progressive disease that affects young men [1]. Currently, it is known that the disease can affect individuals of any age and sex, with predisposing factors frequently present [2]. In fact, Hippocrates had also previously described a type of erysipelas, appearing randomly or after trauma, spreading rapidly, noting that it was most dangerous when found in the genital or pubic regions [3].

Necrotizing soft tissue infections (NSTIs) are characterized by rapid progression, extensive tissue necrosis, and significant systemic toxicity. They are considered surgical emergencies, requiring rapid diagnosis and instant, aggressive surgical treatment [4]. Fournier's gangrene is distinguished from other forms of NSTIs due to its location in the perineal, perianal, and genital regions.

According to American epidemiological data, Fournier’s gangrene accounts for less than 0.02% of hospital admissions, with a pronounced male predominance (ratio = 10:1). Despite significant advancements in diagnostic methods, surgical techniques, and antibiotic treatments, the mortality rate is still high, up to 40% [1].

Fournier’s gangrene, similarly to all NSTIs, is classified into four types based on the microbiological profile. Type I is a polymicrobial infection, Type II involves a monomicrobial infection, most commonly due to Group A β-hemolytic *Streptococcus*, and Type III includes infections caused by *Vibrio* spp., *Aeromonas* spp., or *Clostridium* spp., and Type IV, which includes fungal infections, typically occurring in immunocompromised patients following trauma or burns. Type I is the most frequently observed form of FG [2]. The potential entry points for the pathogenic microorganisms are most commonly the gastrointestinal tract, the genitourinary system, or the skin [1].

As described, the infection is primarily bacterial. Type IV, where fungal pathogens are implicated, is rare, and when it occurs, *Candida albicans* is typically the responsible species [2]. An extremely unusual causal agent is *Candida glabrata*, primarily infecting immunosuppressed patients or those with prior broad-spectrum antibiotic use. *Candida glabrata* presents significant therapeutic challenges due to its decreased susceptibility to azole antifungals. Its isolation in cases of Fournier’s gangrene necessitates prompt antifungal therapy and often indicates underlying immunological compromise [5].

Several factors have been identified as being associated with an increased risk of developing Fournier’s gangrene. Male sex, diabetes mellitus, and a history of alcohol abuse are some of the most strongly correlated [1], whereas in female patients, obesity is considered a significant risk factor, as indicated in several studies [6]. Smoking, malnutrition, immunosuppressive medication, cancer, liver or kidney failure, hypertension, congestive heart failure, and peripheral vascular disease are other risk factors that have been detected [7]. Nevertheless, it is crucial to note that the absence of recognized risk factors does not exclude the possibility of developing the disease. Fournier’s gangrene may also occur in healthy individuals, indicating the need for a high level of suspicion, regardless of the patient's medical history [1].

The disease may be difficult to identify, particularly in the early stages, when lack of symptoms is common (40%). As a result, up to 75% of cases are misdiagnosed and consequently mistreated at first [1]. Some of these cases may result in systemic sepsis, a life-threatening condition defined by extensive inflammation and multi-organ dysfunction [7]. Thus, the ensuing deterioration in health quality further indicates the importance of early recognition and aggressive treatment, as there is evidence suggesting that necrotizing fasciitis can spread at a rate of 2-3 cm/h, providing little time available for intervention [7].

The purpose of this case report is to contribute to the existing literature by presenting one of the few patients with Fournier’s gangrene and only *Candida glabrata* isolated in necrotizing tissue culture. By sharing this clinical experience, we seek to raise awareness and provide guidance for prompt and effective management of this life-threatening condition.

## Case presentation

A 58-year-old female patient presented to the gynecological department, complaining about persistent pain in the vulvar area, especially in the left labia majora, for over a week. Immediate clinical examination revealed inflammation in the area, such as tenderness, erythema, and edema. A large vulvar lesion, resembling condyloma acuminatum, was also detected at the four-to-five o’clock position on the left labia majora.

The patient’s medical history included type two diabetes onset seven years ago (on metformin 1 g twice daily), chronic depression (on vortioxetine 40 mg daily for the last two years), and vestibular neuritis that presented six months ago (on corticosteroid therapy with methylprednisolone, flunarizine, duloxetine, and gabapentin). Surgical history included a cholecystectomy (1993) and umbilical hernia repair with mesh placement (2001). She reported no known drug allergy. Remarkably, a 60-pack-year history of active smoking was reported, as well as a family history of breast cancer in the patient’s sister at age 47.

**Figure 1:**
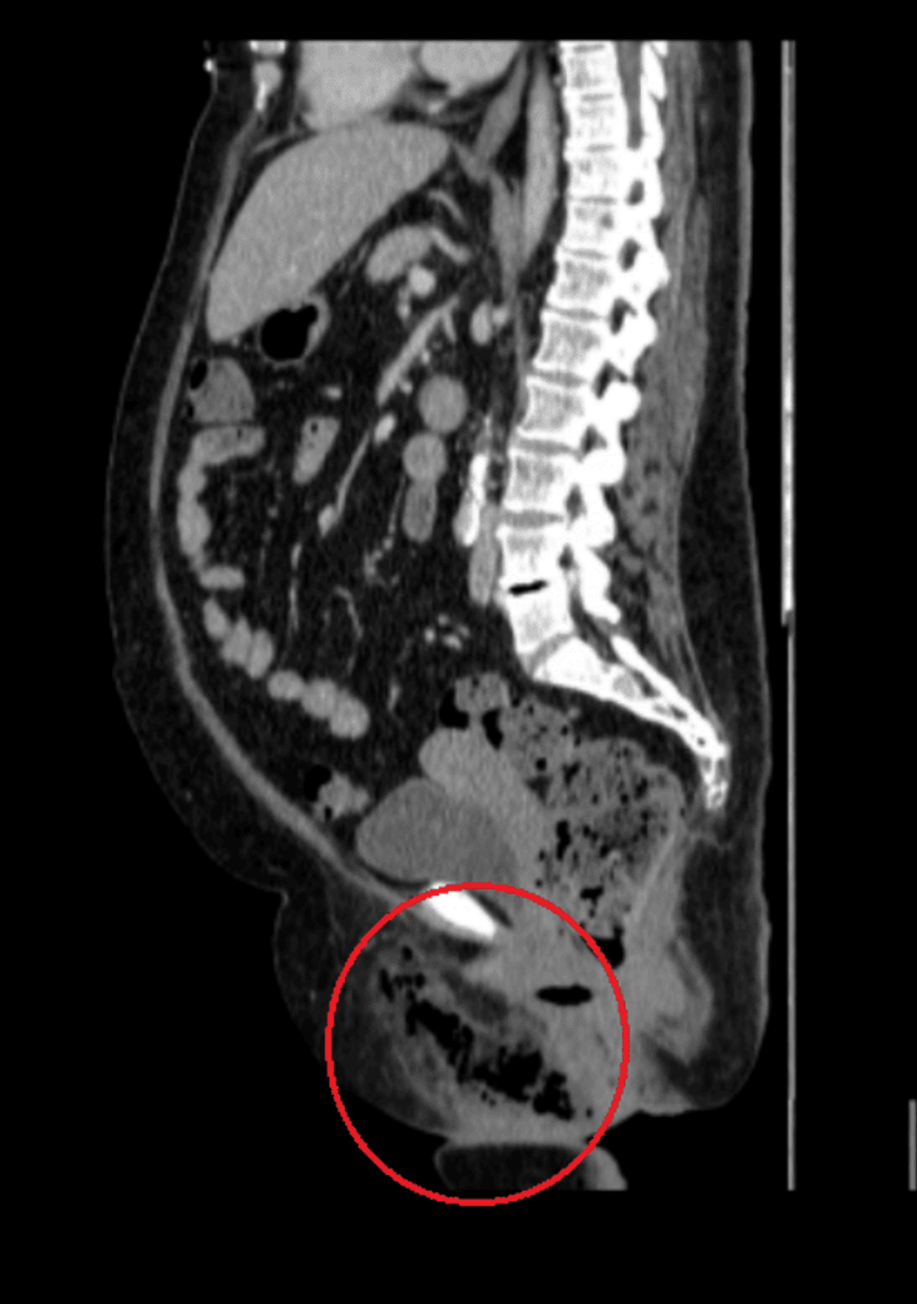
Gas in the subcutaneous tissues of the perineum in contrast-enhanced CT (sagittal plane) CT: computed tomography

**Figure 2:**
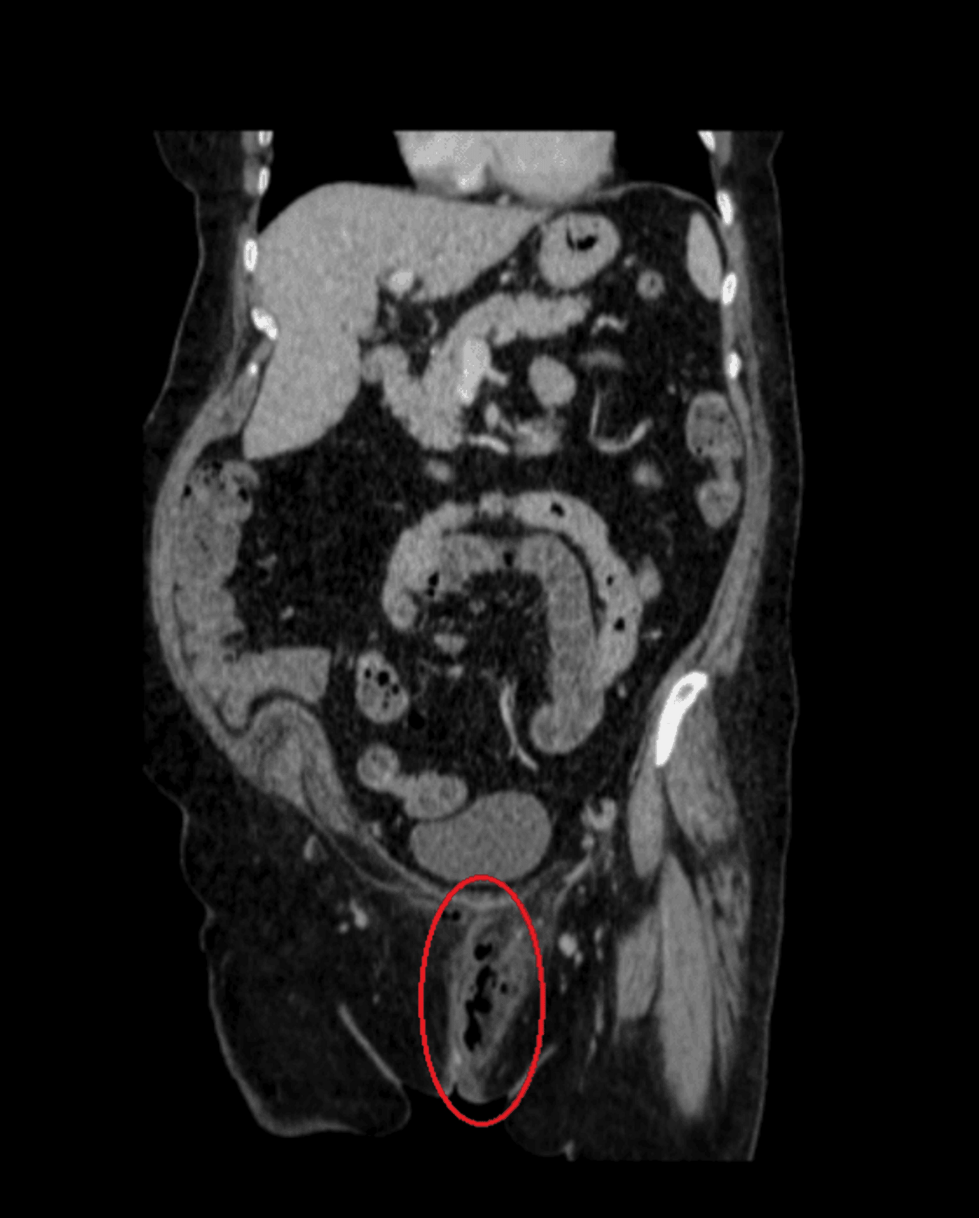
Gas in the subcutaneous tissues of the perineum in contrast-enhanced CT (coronary plane) CT: computed tomography

**Figure 3:**
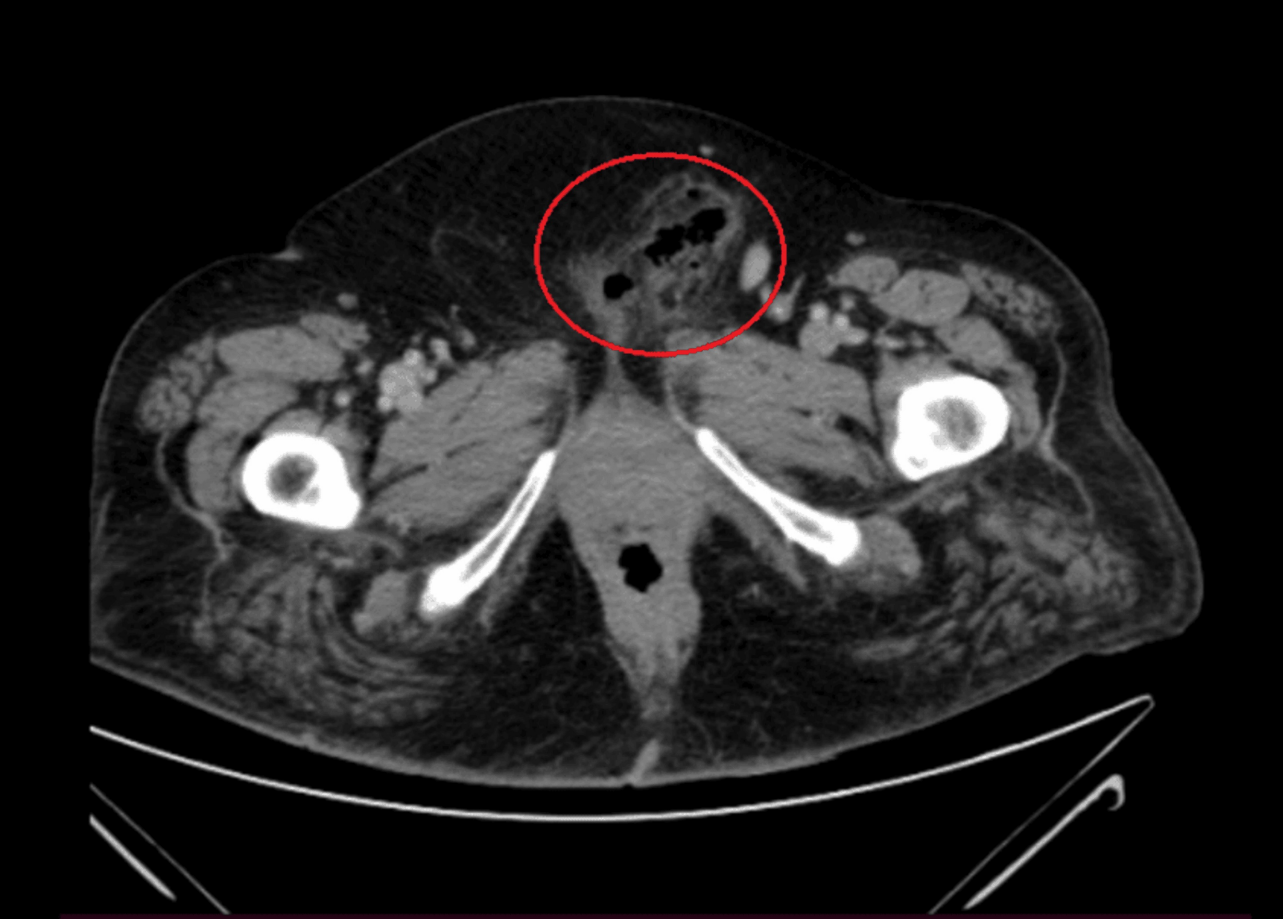
Gas in the subcutaneous tissues of the perineum in contrast-enhanced CT (axial plane) CT: computed tomography

Initially, the patient’s mild clinical presentation contributed to a diagnosis of vulvovaginal infection. As a result, a sample of vaginal fluid was collected for further investigation, and empirical antibiotic therapy, appropriate for the suspected condition, was initiated with amoxicillin-clavulanic acid, doxycycline, and metronidazole. The patient’s symptoms did not improve after 12 hours, and a surgical consultation was requested. Further physical examination revealed a palpable crepitus, which raised concern for NSTI. Along with a novel complete blood count and biochemical laboratory exams (Table 1), imaging with contrast-enhanced CT was urgently requested and confirmed the diagnosis, with the presence of gas in the subcutaneous tissues of the perineum (Figures 1-3). Following the establishment of diagnosis on the same day, the patient was transferred to the 4th Department of Surgery of Attikon University Hospital for further management.

**Table 1:** Laboratory exams

Laboratory exam	Value
Red blood cells	5.12 M/μL
Hemoglobulin	15.4 mg/dL
Hematocrit	45.4%
Platelets	287 K/μL
White blood cells	28.43 K/μL
Neutrophils	25.14 K/μL
Glucose	207 mg/dL
Creatinine	0.92 mg/dL
HbA1c	9.5%
C-reactive protein	97 mg/L
Na^+^	134 mmol/L

**Figure 4:**
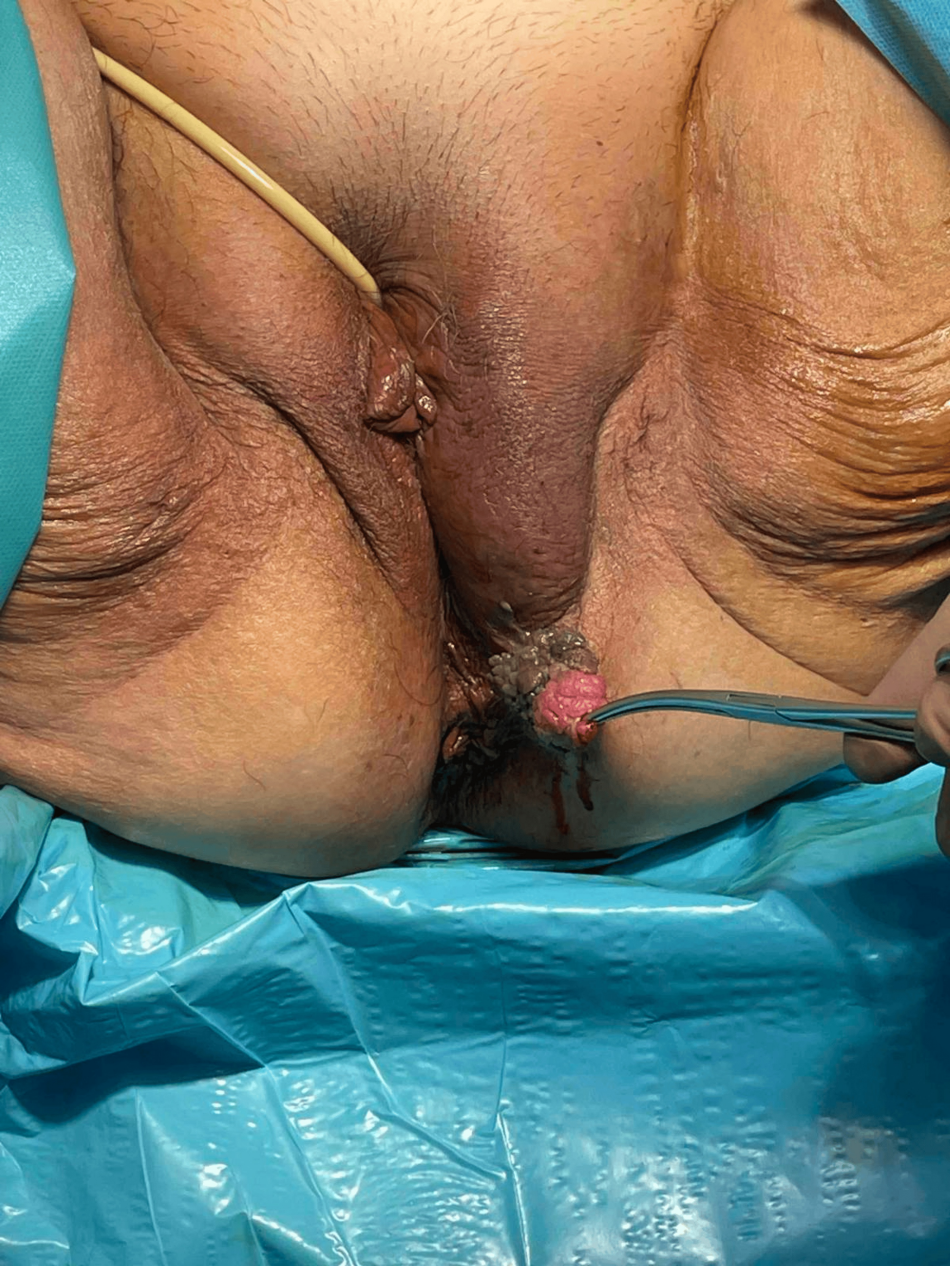
Preoperative image of the patient showing the edema of the left labia major and the condyloma

**Figure 5:**
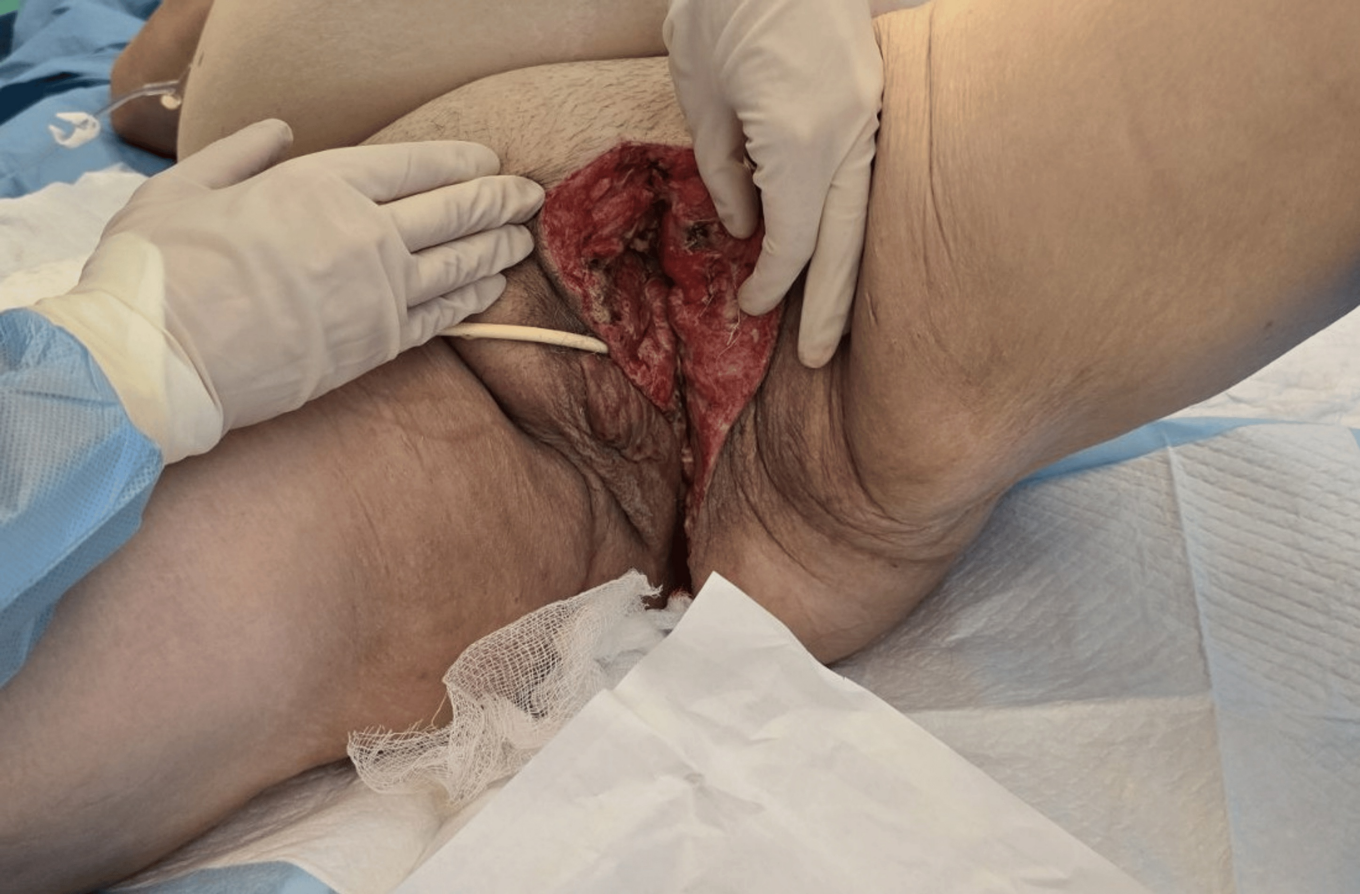
Postoperative image of the patient showing the extent of the excised tissue

The patient underwent emergency debridement of the necrotic tissue alongside excision of the condyloma (Figures 4-5). Empirical preoperative antibiotics consistent with the newly established diagnosis (vancomycin, piperacillin-tazobactam, and clindamycin) were given. Postoperatively, the antimicrobial therapy was escalated due to the culture results from both vaginal fluid and necrotic tissue, which surprisingly revealed only *Candida glabrata*, and the patient received anidulafungin, tigecycline, ciprofloxacin, and metronidazole.

The wound was decided to heal on secondary closure; a vacuum-assisted closure (VAC) device was used. There was no need to re-enter the OR for further excision. After the patient’s clinical improvement, she was discharged on the 32nd postoperative day. Further wound care with regular dressing changes was suggested. On a follow-up examination two months later, the patient was found completely healed with a minimal scar.

The histopathological examination of the excised vulvar lesion confirmed the presence of a condyloma acuminatum, without any evidence of malignancy and a maximal diameter of 4 cm. On follow-up, the wound was adequately closed, and the patient returned to normal activities with no compromise of her quality of life or recurrence of the infection.

## Discussion

The incidence of Fournier’s gangrene in female patients is rare, and the diagnostic procedure is quite challenging due to anatomical and clinical differences, especially when the disease presents with nonspecific symptoms such as localized vulvar pain and inflammation [6,8]. Notably, despite the reduced incidence in women, the mortality rate is higher, ranging from 20% to 50%, compared to 7.5% in males [6]. This case highlights the difficulty of early recognition in women, especially with atypical clinical manifestations, as well as the isolation of *Candida glabrata* as a causative organism.

In this patient, the initial mild clinical presentation contributed to a diagnosis of a vulvovaginal infection, leading to treatment with empirical broad-spectrum antibiotics. Such a diagnostic delay and misdiagnosis for other benign conditions, such as cellulitis, erysipelas, and impetigo, are common. Up to 75% of the cases are misdiagnosed due to the absence of severe skin manifestations and hallmark signs such as crepitus [1]. As a result, treatment, and especially surgical intervention, which is a definitive factor, is delayed, further increasing the already high mortality rate up to 88% [1]. When left untreated, the infection can progress rapidly, leading to extensive soft tissue necrosis and severe complications such as sepsis, acute renal failure, heart failure and arrhythmias, acute respiratory distress syndrome, and finally bacteremia. Bacteremia can, in turn, lead to thromboembolic events in lower limbs and potential amputations [1].

In this patient, several predisposing factors were present, such as diabetes, heavy smoking (reportedly 60 pack-years), immunosuppression due to corticosteroid use, and obesity (95 kg, 1.68 cm, BMI = 33.7 kg/m²), which is considered a deteriorating factor mainly in female patients. Additionally, the presence of a vulvar condyloma may have facilitated the entry of microbial pathogens by compromising the local skin barriers.

The laboratory risk indicator for necrotizing fasciitis (LRINEC) is a clinical scoring system that assists clinicians in differentiating NSTIs from other less malignant soft tissue infections. A score of more than six suggests the presence of an NSTI. Due to its low sensitivity for necrotizing fasciitis, it cannot exclude the diagnosis without clinical and radiographic assessment [9]. That was also the case in our patient, where LRINEC scored low (equal to five, as indicated by the values in Table 2), and as a result, it did not prove to be of aid. The limited practicality of clinical scoring systems, like LRINEC, especially in early and atypical presentations of the disease, underlines the importance of clinical judgment, reinforced by imaging and surgical exploration when there is suspicion of NSTI [9].

**Table 2:** LRINEC LRINEC: laboratory risk indicator for necrotizing fasciitis

Laboratory parameter	Measured value in the patient’s preoperative exam	LRINEC score
C-reactive protein	97 mg/Dl	0
White blood cell count	28.43 per mm^3^	2
Hemoglobin	15.4 gr/dl	0
Sodium	134 mmol/L	2
Creatinine	0.92 mg/dl	0
Glucose	207 mg/dL	1
Total		5

Surgical debridement remains the cornerstone of treatment. Ideally, it should be performed within 24 hours of the patient’s admission, with re-exploration every 12-48 hours if needed. Debridement of all the necrotic tissue should be conducted until healthy tissue is reached, particularly normal fascia rather than skin. Additionally, a tissue biopsy should be obtained for histological analysis. Following this, conventional dressings or a VAC can be used [10]. VAC, the treatment we used for this patient, promotes healing by applying negative pressure, which enhances blood supply, cell migration, and proliferation in the area. Compared to conventional dressings, it is associated with less pain, increased mobilization, and less frequent changes. Later, reconstructive surgeries may be required. Definitive wound closure should be attempted only after infection resolution and granulation tissue formation. Other approaches for secondary wound healing include skin grafts and flap reconstruction with variable techniques [11].

In our patient, aggressive surgical debridement was performed right after confirming the diagnosis with the detection of crepitus in CT imaging. At first, empirical broad-spectrum antibiotics were administered, and later the antibiotic treatment was modified appropriately, according to culture results. Type IV is uncommon, and empirical antibiotic therapy was not accompanied by an antifungal agent.

A particularly remarkable aspect of this case was the isolation of *Candida glabrata* from necrotic tissue cultures. Infection from fungal pathogens in Fournier’s gangrene is significantly rare and typically classified as Type IV NSTIs. When these pathogens are implicated, *Candida albicans* is mostly reported [2]. *Candida glabrata* is a very unusual causal agent, primarily infecting immunosuppressed patients or those with prior broad-spectrum antibiotic use. Septimus et al. published the first case of Fournier’s gangrene due to *Candida glabrata* in 2002 [12], and since then, there have been five additional cases mentioning this pathogen as one of the causative agents for this condition or other necrotizing soft tissue fasciitis [13-17]. A table comparing the management and outcome of all cases of *Candida glabrata* infections that have been cited has been created to provide a complete approach to similar cases (Table 3). *Candida glabrata* is more resistant to azole antifungals; therefore, its presence in necrotic tissue, as in this case, indicates a possibly severe infection with limited therapeutic options [5]. The recommended first-line treatment for *Candida glabrata* infections is echinocandins. Consequently, in this case, early initiation of appropriate antifungal therapy with anidulafungin was essential for a favorable outcome [5,16,18,19]. Although fungal NSTIs are rare, an antifungal agent could be added to the empirical antibiotic therapy in immunocompromised patients who are at risk of this type of infection.

**Table 3:** Comparison of Candida glabrata cases in literature IV: intravenous, ICU: intensive care unit

Title	Authors	Year	Pathogen	Other pathogens	Management	Outcome
Fournier’s gangrene due to *Candida glabrata*: case report and review of the literature [12]	Septimus et al.	2002	Candida glabrata	-	Surgical debridement, preoperative administration of vancomycin and gentamycin, postoperative administration of vancomycin and piperacillin/ tazobactam, and post-culture administration of an increased dose of fluconazole.	All cultures failed to yield any organisms after the initial isolations. The patient died due to heart failure and respiratory difficulties.
Fournier's gangrene due to *Candida glabrata* [13]	Loulergue et al.	2008	Candida glabrata	-	Surgical debridement, initial administration of piperacillin/tazobactam, gentamycin, and aspofungin. After two days of administration of caspofungin only, for 30 days.	Therapy was completed within a month, and the wound was found to have improved.
Necrotizing soft tissue infection of the glans penis due to atypical *Candida* species complicated with Fournier’s gangrene [14]	Jensen et al.	2010	Candida glabrata	*Candida tropicalis*, *Escherichia coli*, *Escherichia faecalis*, mixed anaerobes	Resection of scrotal skin and the left testis. Administration of IV caspofungin, tazobactam, ciprofloxacin, and metronidazole.	Penile lesion improved within five days. Several surgical revisions of the scrotal and perineal areas were performed. Full recovery was achieved.
Necrotizing soft-tissue infection caused by both *Candida glabrata* and *Streptococcus* agalactiae [15]	Shindo et al.	2009	Candida glabrata	Streptococcus agalactiae	After culture of the purulent discharge, administration of IV fluconazole, imipenem, and surgical debridement followed.	After hospitalization for a month, the wound improved, and the patient was discharged.
Gluteal necrotizing soft tissue infection and hip osteomyelitis due to *Candida glabrata* [16]	Henry et al.	2021	Candida glabrata	Mixed enteric flora was isolated, but estimated to be fecal soiling	The patient presented hemodynamically unstable, after drainage and 3 weeks of antibiotic treatment of a suspected perirectal abscess, ICU administration for resuscitation, administration of piperacillin/tazobactam, and inezolid. The patient entered septic shock, and advanced antimicrobial therapy was initiated: piperacillin/tazobactam, clindamycin, vancomycin, fluconazole, and surgical debridement was performed. Postoperative administration of piperacillin/tazobactam and micafungin. Several reoperations were performed with fecal diversion. Negative pressure therapy for 2 months was applied, and wound closure with grafts and flaps.	Patient was discharged after 4 months.
A case of Fournier gangrene caused by *Candida glabrata* [17]	Matsumoto et al.	2014	Candida glabrata	-	Preoperative and postoperative antibacterial agents were administered. Surgical debridement and drainage were performed.	Discharged after 46 days.

This case highlights the importance of maintaining a high level of suspicion for Fournier’s gangrene, even in atypical populations and in the absence of severe clinical manifestations. The limitations of standard risk stratification tools are also underlined, reinforcing the need for a more careful approach in patients with atypical symptoms. Moreover, the necessity of taking into consideration unusual pathogens, including fungal species such as *Candida glabrata*, is indicated, especially in patients with recent extensive antibiotic use or a compromised immune system.

## Conclusions

Fournier’s gangrene is considered a serious surgical emergency due to its aggressive progression and high mortality potential. The isolation of *Candida glabrata* highlights the need to consider rare fungal pathogens, especially in immunocompromised patients, and potentially add broad-spectrum antifungal agents in the empirical treatment. By sharing this unique clinical experience, we aim to raise awareness about the diagnostic challenges, the broadened microbiological spectrum, and treatment strategies for successful outcomes in patients with atypical presentations of this life-threatening condition.
